# 2-(Methylthio) Benzothiazole (MTBT) Induces Cardiovascular Toxicity in Zebrafish Larvae and Investigates Its Mechanism

**DOI:** 10.3390/biology14101398

**Published:** 2025-10-13

**Authors:** Yidi Wang, Junjie Wang, Jie Gu, Fei Ye, Liguo Guo

**Affiliations:** 1 Zhejiang Provincial Center for Disease Control and Prevention, Hangzhou 310051, China; ydwang@cdc.zj.cn; 2 Nanjing Institute of Environmental Sciences, Ministry of Ecology and Environment, Nanjing 210042, China; wangjunjie@nies.org (J.W.); gujie@nies.org (J.G.)

**Keywords:** 2-(Methylthio) benzothiazole (MTBT), zebrafish, cardiovascular toxicity, protein–protein interaction (PPI) network

## Abstract

**Simple Summary:**

In this study, using zebrafish as a model, we found that 2-(Methylthio) benzothiazole (MTBT) decreased heart rate, caused pericardial edema and cardiac malformations, and reduced circulatory function in zebrafish larvae, leading to multiple vascular structural abnormalities. Mechanistic studies showed that MTBT activates the apoptotic pathway by upregulating PTGS2 expression, which in turn mediates cardiovascular development and functional toxicity. This study is the first to systematically reveal the cardiovascular toxicity of MTBT, providing a scientific basis for its environmental safety evaluation and risk management.

**Abstract:**

2-(Methylthio) benzothiazole (MTBT) is widely used in the industrial and pharmaceutical fields, but limited research has been conducted on its aquatic toxicity. In this study, we established a zebrafish model to systematically evaluate its developmental and functional toxicity, focusing on the cardiovascular systems of larvae. The results showed that MTBT significantly reduced heart rate, caused pericardial edema and deformity, delayed cardiac maturation, decreased stroke volume and cardiac output, and led to vascular structural defects. Mechanistically, MTBT upregulated the expression of the core target PTGS2, activated the apoptotic pathway, and mediated cardiovascular toxicity. This study is the first to systematically confirm the cardiovascular toxicity of MTBT, supplementing its toxicological database and providing a scientific basis for the establishment of environmental safety thresholds and risk management.

## 1. Introduction

Benzothiazoles (BTHs) are widely used as rubber vulcanization accelerators and stabilizers due to their excellent antioxidant, anti-aging, and bacteriostatic properties; they are also used as biocides in wastewater treatment processes [1]. In recent years, some BTH derivatives have been found to have therapeutic potential for Parkinson’s and other neurodegenerative diseases [2]. As the scale of their production and use increases, the detected amounts of BTHs in water bodies worldwide have also increased, entering the aquatic environment through pathways such as surface runoff and industrial and domestic wastewater discharge [3,4]. The available monitoring data show that the average concentration of BTHs in groundwater and surface water is as high as 406 ng/L in 51 Chinese cities [5]. 2-(Methylthio) benzothiazole (MTBT) is one of the main derivatives of 2-mercaptobenzothiazole (MBT) [6]. It is used as an additive to significantly enhance the durability of rubber products [7] and is also a key pharmaceutical intermediate in the synthesis of central nervous system drugs such as antidepressants [8]. Similar to its parent compound, MTBT mainly enters water bodies through rainwater runoff and sewage discharge [3] and has been frequently detected in surface water, groundwater, and wastewater in many regions around the world. For example, MTBT concentrations as high as 4170 ng/L have been detected in surface runoff in Dongguan and Huizhou, China, compared to 738 ng/L in the main streams of the Pearl River and Dongjiang River [9]. In Tianjin, the detection rate of MTBT was as high as 85%, with a peak value of 13.0 μg/L; the highest concentration in sewage reached 31.7 μg/L [10]. MTBT was also detected in underground drinking water sources in Zealand, Denmark [11]. These results highlight MTBT’s widespread contamination of the aquatic environment and the need for a systematic assessment of its environmental risks and health effects.

The above studies indicate that MTBT is widely present in both surface water and underground drinking water, making it a potential threat to the health of aquatic organisms and the balance of aquatic ecosystems, and even to human health. However, there are few studies on its toxicity to aquatic organisms, aside from one study demonstrating the acute toxicity of MTBT to *Ceriodaphnia dubia* [12]. Previous studies have shown that 2-aminobenzothiazole (2-ABTH), which is also a BTH, can cause cardiac developmental malformations in zebrafish, interfering with tubular annulation and decreasing blood flow [13], suggesting that BTHs may have a detrimental effect on the cardiovascular development of living organisms.

Zebrafish (*Danio rerio*) are widely used as a mature aquatic biological test model for drug toxicity screening [1,14,15] and cardiovascular diseases due to their high homology with human genes [16], large spawning capacity, rapid development, and transparent embryos. The latter also improves the ease of observation. With the wide application of transgenic fish such as *Tg(fli1:eGFP)* and *Tg(myl7:eGFP)*, it has become possible to observe their cardiovascular development more directly [17]. In this study, using an experimental zebrafish model, we investigated the effects and potential mechanism of action of early MTBT exposure on the cardiovascular development and function of zebrafish larvae, aiming to provide support for the rational control and risk assessment of MTBT with this toxicological data.

## 2. Materials and Methods

### 2.1. Drugs and Reagents

MTBT (CAS No. 615-22-5, >97% purity) was purchased from McLean Reagent (Shanghai, China). Dimethyl sulfoxide (DMSO, purity > 99.7%) and Trizol were purchased from Sigma-Aldrich Corporation ((St. Louis, MO, USA). The cDNA reverse transcription kit and SYBR^®^ Green Master Mix kit were purchased from Nanjing Novozymes Bio-technology Co., Ltd. (Nanjing, China). PCR primers were synthesized by Shanghai Jierui Bioengineering Co., Ltd. (Shanghai, China).

### 2.2. Zebrafish Culture and Embryo Acquisition

The fish used in the experiment included the wild-type AB line, cardiomyocyte GFP-labeled *Tg(myl7:eGFP)* line, and vascular endothelial cell GFP-labeled *Tg(fli1:eGFP)* line, which were initially purchased from the National Zebrafish Resource Center (Wuhan, China). They were kept in a recirculating water system in the laboratory under the following conditions: water temperature 27.0–28.0 °C, conductivity 480–510 µS cm^−1^, pH 7.0–7.5, and a photoperiod of 14 h of light/10 h of darkness. Prior to the exposure test, 20 healthy adult males and 20 females were randomly selected and preconditioned for 7 d to confirm good reproductive status. In the evening of the pairing day, the animals were placed in a breeding box with a 2:1 ratio of males to females, separated by a transparent partition; the morning of the next day, the partition was removed, and the light was turned on to induce spawning. The embryos were collected 2 h post fertilization (hpf) and incubated at 27.5 °C in a constant temperature incubator. Incubation was continued for 2 h. Embryos at the 4 hpf stage were screened under a stereomicroscope for normal development and used in subsequent exposure experiments. All zebrafish experiments were approved by the Animal Ethics Committee of the Nanjing Institute of Environmental Science (Approval No. 20241017) (Table S1).

### 2.3. Solution Preparation and Exposure Test Design

The MTBT exposure solution was prepared using stepwise dilution: 10 mg of the original drug was dissolved in DMSO and concentrated to 1 mL to obtain 10,000 mg/L of stock solution; 0.1 mL of the solution was added to 0.9 mL of DMSO and mixed to obtain 1000 mg/L of secondary stock solution; then, the solution was diluted stepwise with culture water according to the target concentration. Six groups (0, 1, 2, 4, 8, and 16 mg/L) were set up for acute exposure according to the pre-test. Five groups (0 (CK), 1/1000 LC_50_, 1/100 LC_50_, and 1/10 LC_50_) were set up for the acute LC_50_ test to measure developmental and cardiovascular toxicity; their concentrations were set at 0, 15, 150, and 1500 μg/L, respectively. Three (biological) parallels were set for each concentration, and 6-well plates were used as the exposure containers. A total of 5 mL of exposure solution was added to each well, and ten 4 hpf embryos were placed in each well; death, incubation, and hatching conditions were recorded on a daily basis. Dead individuals were removed, and the total exposure time was 72 h.

### 2.4. Developmental Toxicity Assessment of Zebrafish Larvae

After 72 h of MTBT exposure, 12 zebrafish larvae (4 per parallel) were randomly taken from each group, fixed with 3% methylcellulose, and placed under a body microscope (Nikon SMZ25, Nikon, Tokyo, Japan) to collect bright-field images along with 30 s videos of the cardiac region. Images were imported into NIS-Elements D v5.41.00 software to measure body length, eye area, and pericardial area; videos were imported into DanioScope (Noldus, Wageningen, The Netherlands) to quantify heart rate.

### 2.5. Assessment of Cardiac Development and Function in Zebrafish Larvae

After 72 h of MTBT exposure, 12 *Tg(myl7:eGFP)* transgenic juvenile fish were randomly selected from each group, fixed with 3% methylcellulose and repositioned to acquire cardiac fluorescence images and videos using a fluorescence body microscope (Nikon SMZ25, Nikon, Tokyo, Japan): cardiac fluorescence images were acquired in the lateral position, and then videos of the heartbeats were recorded for 20 s in the supine position. The fluorescence images were measured using NIS-Elements D v5.41.00 software to measure the straight-line distance from the sinus venosus (SV) to the bulbus arteriosus (BA) to assess cardiac development. The video was analyzed frame by frame to determine the longitudinal axis length (a) and transverse axis length (b) of the ventricle at end-systole and end-diastole; end-diastolic volume (EDV), end-systolic volume (ESV), stroke volume per beat (SV), and cardiac output (CO) were also analyzed to quantify cardiac pumping function [17,18].

### 2.6. Assessment of Vascular Development in Zebrafish Larvae

After 30, 48, and 72 h of MTBT exposure, images of the intersegmental vessels (ISVs) at 30 hpf, the main vein (CCV) at 48 hpf, and the subintestinal vessels (SIVs) at 72 hpf were sequentially captured with a fluorescence somatic microscope (Nikon SMZ25, Japan); the total length of the ISVs and the complete rate were subsequently measured with NIS-Elements D v5.41.00, and the areas of the CCVs and SIVs were calculated separately to systematically assess the process of vessel development.

### 2.7. Gene Expression Measurements

After 72 h of MTBT exposure, 150 zebrafish larvae were randomly selected from each group (3 parallels, 50 fish in each parallel) for gene expression analysis. The samples were homogenized with Trizol (1 mL) to extract total RNA, and the concentration was determined with a NanoDrop™ 2000 Ultra-Micro Spectrophotometer (Thermo Fisher Scientific, Madison, WI, USA) and diluted to 200–400 ng/μL; then, cDNA was synthesized using a reverse transcription kit. Real-time fluorescence quantitative PCR was performed on a CFX Connect™ system (Bio-Rad, Hercules, CA, USA), and the reaction system consisted of SYBR^®^ Green Master Mix, cDNA, and corresponding primers. The relative expression was calculated using the 2^−ΔΔCt^ method [14], and the primer sequences used are listed in Table 1.

### 2.8. Protein–Protein Interaction (PPI) Network Analysis

Firstly, the Simplified Molecular Input Line Entry System (SMILES) number of MTBT was retrieved from the PubChem website (https://pubchem.ncbi.nlm.nih.gov/) and entered into the SwissTargetPrediction website (http://swisstargetprediction.ch/) to predict its potential targets of action; subsequently, the GeneCards database (https://www.genecards.org/) was used to search for genes related to cardiovascular toxicity and analyze any intersection with the former. Finally, PPI networks of the proteins encoded by the above intersecting genes were constructed on the STRING platform (https://cn.string-db.org/). In order to further elucidate the potential mechanism of MTBT-induced cardiovascular toxicity, the intersected genes were also input into the online microbiology platform (https://www.bioinformatics.com.cn/) to conduct pathway enrichment analyses for the Kyoto Encyclopedia of Genes and Genomes (KEGG) and Gene Ontology (GO) databases.

### 2.9. Statistics and Analysis of Data

For developmental and cardiovascular toxicity indicators, 12 observations were randomly collected from each treatment group, and 10 valid data points were retained for statistical purposes after excluding the maximum and minimum extremes. All data were statistically analyzed and visualized using GraphPad Prism 8.0, and the results are expressed as mean ± standard error of the mean (SEM). Differences between groups were analyzed by one-way ANOVA with Dunnett’s multiple comparisons test, and the significance level was set at *p* < 0.05. Significance markers: *p* < 0.001 (***), *p* < 0.01 (**), and *p* < 0.05 (*).

## 3. Results

### 3.1. Effects of MTBT Exposure on the Early Development of Zebrafish Larvae

**Figure 1:**
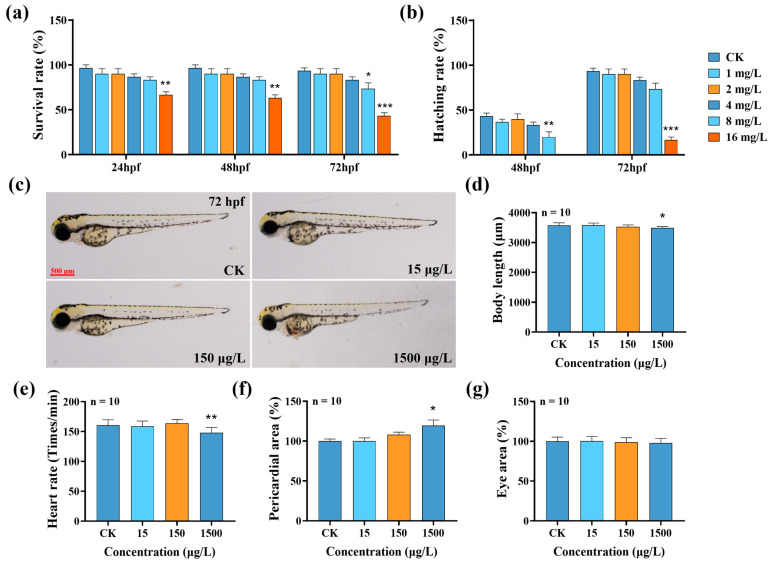
Effect of MTBT exposure on the early development of zebrafish larvae. Statistical graphs of (**a**) survival and (**b**) hatching rates of zebrafish larvae after 72 h of MTBT exposure. (**c**) Typical images of zebrafish larvae after 72 hpf. Statistical plots of larval (**d**) body length, (**e**) heart rate, (**f**) pericardial area, and (**g**) eye area. *p* values: *p* < 0.001 (***), *p* < 0.01 (**), and *p* < 0.05 (*).

The results of the 72 h acute toxicity test showed that the 8 and 16 mg/L MTBT treatments significantly reduced the survival and hatchability of zebrafish larvae compared with the control (Figure 1a,b, *p* < 0.05); the 72 h LC_50_ was calculated as 16.11 mg/L. Developmental toxicity assessment further showed that the 1500 μg/L MTBT treatment significantly reduced body length and heart rate by 2.21% and 7.80%, respectively (Figure 1d,e, *p* < 0.05), while the pericardial area was significantly increased by 34.34% (Figure 1f, *p* < 0.05), whereas the eye area did not show any significant change (Figure 1g, *p* > 0.05). In conclusion, acute exposure to MTBT can cause significant early developmental damage to zebrafish larvae.

### 3.2. Effects of MTBT Exposure on Cardiac Development and Function in Zebrafish Larvae

**Figure 2:**
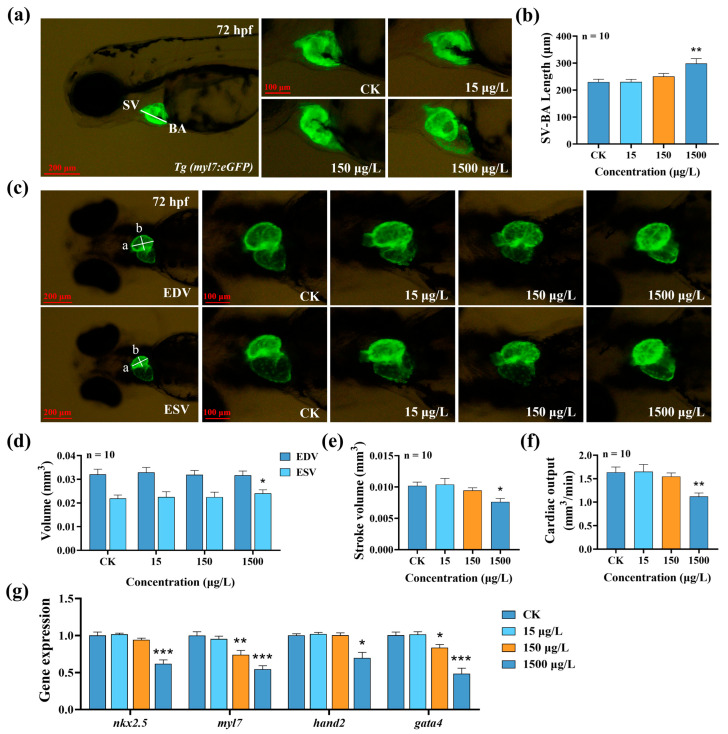
Effects of MTBT exposure on heart development and function in zebrafish larvae: (**a**) Typical fluorescence images of heart development in *Tg(myl7:eGFP)* zebrafish larvae after 72 h of MTBT exposure, and (**b**) statistical graph of SV-BA length. (**c**) Typical fluorescence images of ventricular end-diastole and end-systole in *Tg(myl7:eGFP)* zebrafish larvae after 72 h of MTBT exposure, and statistical graphs of (**d**) EDV and ESV, (**e**) SV, and (**f**) CO in zebrafish larvae. (**g**) Statistical maps of expression of cardiac development-related genes (*nkx2.5*, *myl7*, *hand2*, and *gata4*) in zebrafish larvae. *p* values: *p* < 0.001 (***), *p* < 0.01 (**), and *p* < 0.05 (*).

Since our developmental toxicity test suggested that MTBT can induce pericardial edema and reduce heart rate, we further evaluated the cardiotoxicity of *Tg(myl7:eGFP)* transgenic lines. The results showed that the 1500 μg/L MTBT treatment significantly prolonged the SV-BA distance (Figure 2a,b, *p* < 0.01), indicating that cardiac cyclisation was blocked and development was delayed; at the same time, the expression levels of early cardiac development-related genes (*nkx2.5*, *myl7*, *hand2*, and *gata4*) were significantly downregulated (Figure 2g, *p* < 0.05), further verifying that *Tg(myl7:eGFP)* can induce pericardial oedema and decrease heart rate at the molecular level.

Our assessment of cardiac pumping function showed that 1500 μg/L MTBT exposure did not significantly alter EDV, but significantly increased ESV (Figure 2d, *p* < 0.05), which led to a significant SV reduction of 25.12% (Figure 2e, *p* < 0.05); coupled with the decrease in heart rate during the same period of time, a significant decrease in CO was observed (Figure 2f, *p* < 0.01). These results consistently show that acute exposure to MTBT significantly impaired the pumping function of the heart in zebrafish larvae.

### 3.3. Effects of MTBT Exposure on Vascular Development in Zebrafish Larvae

**Figure 3:**
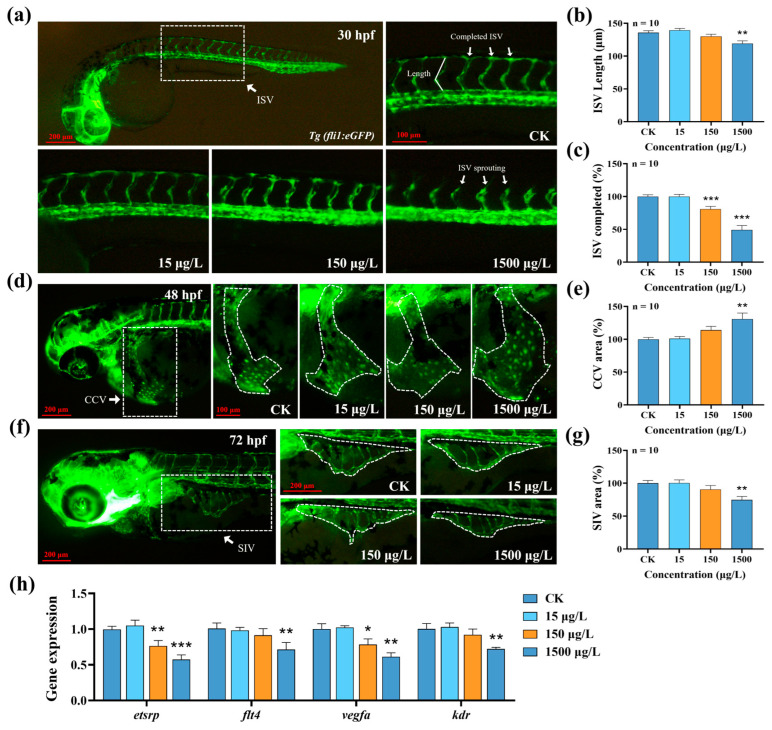
Effect of MTBT exposure on vascular development in zebrafish larvae. (**a**) Typical fluorescence images of ISV in 30 hpf *Tg(fli1:eGFP)* transgenic zebrafish larvae, ISV (**b**) length, and (**c**) completed rate statistic plots. (**d**) Typical fluorescence image of CCV in 48 hpf *Tg(fli1:eGFP)* transgenic zebrafish larvae and (**e**) statistical graph. (**f**) Typical fluorescence images of SIV in 72 hpf *Tg(fli1:eGFP)* transgenic zebrafish larvae and (**g**) statistical graph. (**h**) Statistical graph of the expression of vascular development-related genes (*etsrp*, *flt4*, *vegfα*, and *kdr*). *p* values: *p* < 0.001 (***), *p* < 0.01 (**), and *p* < 0.05 (*).

To further clarify the acute effects of MTBT on the vascular system, we systematically evaluated the development of ISV, CCV, and SIV using the *Tg(fli1:eGFP)* transgenic zebrafish as a model. The results showed that after 30 h of exposure, 1500 μg/L MTBT significantly shortened the length of ISVs (Figure 3b, *p* < 0.01), and the 150 and 1500 μg/L treatments decreased the anastomosis rates of ISVs by 19.13% and 50.75%, respectively (Figure 3c, *p* < 0.001). After 48 h and 72 h of exposure, the CCV area of zebrafish larvae in the 1500 μg/L group was significantly enlarged, while the SIV area was significantly reduced (Figure 3d–g, *p* < 0.01). In addition, the expression levels of key genes for vascular development (*etsrp*, *flt4*, *vegfα*, and *kdr*) were significantly downregulated in 72 hpf zebrafish larvae (Figure 3h, *p* < 0.05). In conclusion, acute exposure to MTBT significantly inhibited the normal development of the early vascular system in zebrafish.

### 3.4. Potential Mechanism of Action of MTBT in Inducing Cardiovascular Toxicity in Zebrafish Larvae

**Figure 4:**
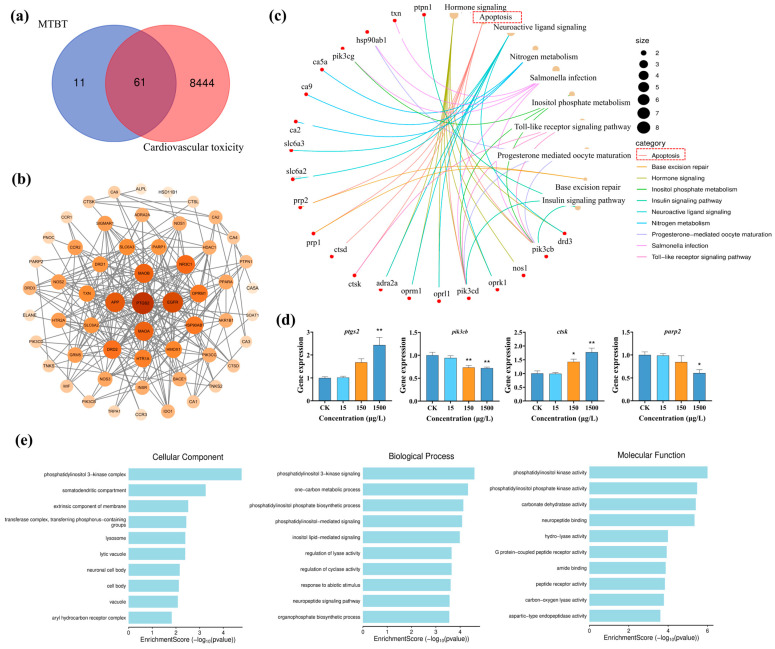
Potential mechanism of action of MTBT in inducing cardiovascular toxicity in zebrafish larvae. (**a**) Number of target genes at the intersection of MTBT and cardiovascular toxicity. (**b**) PPI network analysis diagram. (**c**) Graph of the results of KEGG enrichment analysis of intersecting genes. (**d**) Graph of expression statistics of *ptgs2* and apoptotic pathway genes (*pik3cb*, *ctsk*, and *parp2*). (**e**) Graph of the results of the enrichment analysis of the intersecting gene GO. *p* values: *p* < 0.01 (**), and *p* < 0.05 (*).

To elucidate the molecular mechanism of MTBT-induced cardiovascular toxicity in zebrafish larvae, we integrated the PPI network and multi-omics enrichment analyses. Firstly, 61 intersecting MTBT–cardiovascular toxicity target genes were obtained by integrating SwissTargetPrediction and GeneCards data (Figure 4a), and our topological analysis of the PPI network showed that prostaglandin endoperoxide synthase 2 (PTGS2) was the most highly connected core protein (Figure 4b). This indicates that PTGS2 could be a key node in MTBT-mediated cardiovascular injury. KEGG enrichment showed that the intersecting genes were significantly enriched in the “apoptosis” pathway (Figure 4c). qPCR confirmed that the expression levels of *ptgs2* and downstream apoptosis-related genes (*pik3cb*, *ctsk*, and *parp2*) were significantly altered in zebrafish larvae after exposure to 1500 μg/L MTBT (Figure 4d, *p* < 0.05), suggesting that MTBT activates the apoptotic pathway via PTGS2 and induces cardiovascular toxicity. GO enrichment further showed that the intersecting genes were significantly associated with phosphatidylinositol kinase (Figure 4e).

## 4. Discussion

With the rapid expansion of the rubber industry, MTBT has become one of the most common and high-dose benzothiazole rubber additives. It is one of the most frequently detected additives in the environment, as it can continuously enter water bodies through pathways such as surface runoff and wastewater discharge [7,19]. However, aquatic toxicological data for MTBT are still extremely scarce, significantly limiting the potential for ecological risk assessments and control. In order to fill this gap, we used zebrafish as an experimental model, aiming to systematically evaluate the developmental, cardiovascular, and functional toxicity caused by exposure to non-lethal doses, with the aim of providing a scientific basis for the development of environmental safety thresholds.

Acute test results showed that exposure to ≥8 mg/L MTBT significantly reduced the survival and hatching rates of zebrafish larvae, and at a dose of 1500 μg/L, which did not cause survival or hatching abnormalities, MTBT still significantly inhibited body length, reduced heart rate, and induced significant pericardial edema. Pericardial edema is widely regarded as an early indication of impaired cardiac cyclization and pumping insufficiency [20], whereas a decrease in heart rate directly results in a sharp reduction in cardiac output [21]. Together, these results suggest that acute exposure to MTBT can have a dual adverse effect on the morphogenesis and functional performance of the zebrafish heart. Given the fundamental role of the cardiovascular system in embryonic development and nutrient transport [17], MTBT-induced cardiac defects may pose a potential threat to the subsequent growth of individuals and even the continuation of the population.

When normal zebrafish embryos reach 72 hpf, cardiac ring formation and atrium and ventricle differentiation are complete. Quantitative parameters such as heart rate, SV-BA interval, pericardial area, and cardiac output can stably and sensitively reflect early cardiac toxicity [22,23,24]. To further validate its adverse effects, the *Tg(myl7:eGFP)* transgenic line was exposed to MTBT for 72 h to quantitatively assess its impact on the morphology and pumping function of larval hearts. The results showed that exposure to 1500 μg/L MTBT significantly prolonged the SV-BA distance, which is a commonly used parameter for measuring the completion of cardiac cyclization in zebrafish, an increase in which is directly suggestive of impaired cardiac cyclization [25]. At the functional level, MTBT exposure significantly increased ESV and decreased SV and CO, suggesting that it not only interfered with cardiac morphogenesis but also directly impaired pumping ability. Consistent with this, the expression of the key cardiac development genes *nkx2.5*, *myl7*, *hand2*, and *gata4*, which jointly constitute the core regulatory network for heart development, was significantly downregulated. Specifically, Nkx2.5 serves as an early marker for myocardial development and is crucial for maintaining the proliferation of myocardial precursor cells. Reduction in its expression can lead to ventricular dysplasia and pericardial edema [26,27]. Hand2 is a key factor in the process of ventricular morphogenesis, and its abnormal expression directly causes cardiac looping disorders [28,29]. Gata4 is not only indispensable for maintaining the survival of ventricular myocytes but also forms a positive feedback loop with Nkx2.5 to jointly regulate heart development; its functional deficiency is closely related to heart failure [30]. Myl7, as a core gene in atrial myofiber assembly and contraction function, directly leads to atrial contraction weakness if its expression is reduced, thereby causing reduced venous return [31,32]. In conclusion, acute exposure to MTBT caused dual structural–functional damage to zebrafish larvae hearts.

In order to systematically evaluate the effect of MTBT on early vasculogenesis in zebrafish, we dynamically and quantitatively analyzed the developmental processes of the ISVs, CCV, and SIVs using the *Tg(fli1:eGFP)* transgenic strain as a model. The ISV completes anastomosis with the dorsal aorta (DLAV) at 30 hpf, forming a complete dorsal vascular network [33]. At 48 hpf, the endothelial cells of the CCV migrate and gradually lumenize from a lamellar structure, ultimately converging into the cardiac circulatory system [34], while at 72 hpf, the SIV assumes the functions of nutrient transport and blood circulation, forming the key vascular network to maintain the normal growth of zebrafish larvae [35]. The experimental results showed that 1500 μg/L MTBT exposure significantly inhibited the length and anastomosis rate of ISVs in 30 hpf zebrafish larvae, abnormally enlarged the area of CCVs in 48 hpf zebrafish larvae, and significantly reduced the area of SIVs in 72 hpf zebrafish larvae; at the same time, the expression of core genes of vascular development, such as *etsrp*, *flt4*, *vegfα*, and *kdr*, was significantly downregulated. The ETS transcription factor Etsrp is a core regulatory factor in the process of angiogenesis. It can directly activate the gene expression of key receptors such as Flt4 and Kdr, thereby initiating angiogenesis at the transcriptional level [36,37]. Studies have shown that the loss of Etsrp function leads to severe obstruction in the formation of vascular endothelial cells, which in turn affects the development of the entire vascular system [38]. At the level of signal execution, Vegfa, as the most important growth factor, binds to its main receptor Kdr; activates downstream signaling pathways, directly driving the proliferation, migration, and survival of endothelial cells; and is the “executor” of the angiogenesis process [39,40,41]. It is worth noting that Flt4, as another important receptor tyrosine kinase, collaborates with Kdr during early embryonic development to participate in the initial formation and remodeling of blood vessels [42]. The above results consistently showed that acute exposure to MTBT could lead to delayed early vascular development in zebrafish.

PPI networks have been widely used in drug target screening and mechanism analysis [43,44] to predict direct or indirect binding sites between proteins based on molecular structure. In this study, we constructed a PPI network and found that there were 61 intersection nodes between MTBT targets and cardiovascular toxicity-related genes; KEGG enrichment suggested that the “apoptosis pathway” was the most significant, and changes in the expression of key genes of this pathway, such as *pik3cb*, *ctsk*, and *parp2*, further confirmed that MTBT induced cardiovascular toxicity through the apoptosis pathway. Downregulation of PIK3CB inhibits the PI3K/AKT/mTOR axis, leading to the blockage of cardiomyocyte proliferation and the promotion of apoptosis [45,46,47], and its deletion also impairs cardiac function by decreasing the activity of YAP [48] and enhances autophagy-mediated apoptosis of vascular smooth muscle cells (VSMCs) [49]. Cathepsin K (Ctsk) upregulation activates NF-κB signaling and induces apoptosis [50,51,52]; PARP2 inhibition impairs NF-κB regulation and amplifies TNF-α-mediated cell death [53,54,55,56]. The above mechanism is consistent with a previous report that 2-hydroxybenzothiazole (OTH) triggers apoptosis via P53 [57], which further supports the common mechanism that benzothiazoles lead to cardiovascular injury through activation of the apoptotic pathway. PPI network analysis identified that PTGS2 (also known as COX-2) is the core target of MTBT and has the highest association with cardiovascular toxicity, and it directly interacts with key proteins such as PIK3CB, CTSK, and PARP2. As a member of the cyclooxygenase family, PTGS2 can directly activate Caspase-9 to trigger the mitochondrial apoptotic pathway [58]. Its upregulated expression not only exacerbates cardiomyocyte inflammatory response and promotes apoptosis [59], but has also been shown to be a direct driver of atherosclerosis (AS) development and vascular dysfunction [60,61]. All these results suggest that MTBT may lead to early cardiovascular toxicity in zebrafish larvae, with the potential pathway of activation being PTGS2-induced apoptosis.

## 5. Conclusions

In conclusion, the developmental and cardiovascular toxicity of MTBT, an important derivative of BTHs, was systematically evaluated in this study through 72 h acute exposure, using zebrafish larvae as the model organisms. The results showed that environmentally relevant concentrations of MTBT inhibited body length growth and induced pericardial edema, significantly delayed cardiac annulation and vascular network formation, and led to significant impairment of cardiac pumping function. With the help of PPI network analysis, it was further found that the core target of MTBT was PTGS2 (COX-2), which can upregulate the expression of key genes of the apoptosis pathway (*pik3cb*, *ctsk*, and *parp2*) through regulating PI3K/AKT, NF-κB, and other signaling axes, exacerbating the apoptosis of myocardial and vascular endothelial cells and mediating the development of cardiovascular toxicity in zebrafish larvae. However, this study is based solely on PPI network analysis of targets and does not include RNA sequencing. In the future, it is necessary to integrate transcriptome sequencing to further verify the core regulatory network.

## Figures and Tables

**Table 1:** The primer sequences for QPCR.

Accession Number		Forward	Reverse
*NM_181601.5*	β-actin	5′-ACAGGGAAAAGATGACACAGATCA-3′	5′-CAGCCTGGATGGCAACGTA-3′
*NC_133189.1*	nkx2.5	5′-ACAAACATCACTTTGCCGCC-3′	5′-ATGCCCGACTGTTAAGGCTC-3′
*NM_131329*	myl7	5′-TGAGCAATCACAAATACAGG-3′	5′-GCAGCAGTTACAGACAGAATA-3′
*NM_131626.3*	hand2	5′-ACGTCGACAGGGATTGGAAC-3′	5′-CAGACACCAACTGTCTCCCC-3′
*NM_131236*	gata4	5′-TCCAGGCGGGTGGGTTTATC-3′	5′-TGTCTGGTTCAGTCTTGATGGGTC-3′
*NM_001037375.1*	etsrp	5′-GCAGTGGTGAAGACCTGTCC-3′	5′-CAGCCATCACCAGTCCAACT-3′
*NM_130945.2*	flt4	5′-ACCACTATCCCTGAAGGATTCG-3′	5′-GACAGCCACTGTGTTTGCAG-3′
*NC_133191.1*	vegfα	5′-ATGAGAACCACACAGGACGG-3′	5′-ACACTCTCGCTTTGCTTCCT-3′
*NM_001024653.2*	kdr	5′-TGAGAAGGACGTGTCGTTGG-3′	5′-CGTCCACAGAGATACGAGCC-3′
*NM_153657.1*	ptgs2	5′-GCTACAAAAGCTGGGAAGCG-3′	5′-GGTGAAGGCTGGTCCCTTTT-3′
*XM_005157466.6*	pik3cb	5′-TGTGTTCTCTGCCATGCCTC-3′	5′-ACTGGTCGTAGTGCATGGTG-3′
*NC_133191.1*	ctsk	5′-GACCCGTGTCAGTTGGCATA-3′	5′-ACCCCAGCTTCAAAAACAGAAA-3′
*NM_001204270.1*	parp2	5′-CCCCAGTTGTGCTCTGTGAT-3′	5′-CAGGGGCTTTGCCCTTCATA-3′

## Data Availability

The raw data supporting the conclusions of this article will be made available by the authors upon request.

## Supplementary Materials

The following supporting information can be downloaded at: https://www.mdpi.com/article/10.3390/biology14101398/s1. Table S1: Affidavit of Approval of Animal Ethical and Welfare.
