# Supplementary material for: 2-(Methylthio) Benzothiazole (MTBT) Induces Cardiovascular Toxicity in Zebrafish Larvae and Investigates Its Mechanism
Source: Biology (Basel). 2025 Oct 13;14(10):1398. doi: 10.3390/biology14101398 (PMC12561586; doi:10.3390/biology14101398)
Supplement: Supplementary file 1 [file biology-14-01398-s001.zip › biology-3894936-supplementary.pdf]

Supplementary materials

Supplementary Table S1. Affidavit of Approval of Animal Ethical and Welfare.

Affidavit of Approval of Animal Ethical and Welfare

Approval No.	IACUC-20241017

The animal use protocol listed below has been reviewed and approved by the Animal Ethical and Welfare Committee (AEWC), hereby certify.

Protocol Title	Toxicity of 2-(Methylthio)benzothiazole (MTBT) to Zebrafish and Its Potential Mechanism Investigation				
Applicant	Jie Gu	Title/Degree	Assistant researcher	Email	gujie@nies.org
Principle Investigator (PI)	Guixiang Ji	Title/Degree	Researcher	Email	jgx@nies.org
Institution	Nanjing Institute of Environmental Sciences, Ministry of Ecology and Environment				
Species or Strains	Zebrafish (*Danio rerio*)			Quantity	1000 embryos
Period of Protocol	2024/10/17 — 2025/10/17		Application date	2024/10/17	
Number of Animal use permit	SYXK（Su）：2024-10-17				
Results of inspection	☑ Agree.				
Chief Facility Officer	Ge Feng		Date	2024-10-17	
Supplement		Stamp: 农药环境评价与污染控制重点实验室			
